# Design and Initial Psychometric Evaluation of the Multidimensional School Social Climate Inventory for Educational Assistants (MSSCI-EA) in Chile

**DOI:** 10.3390/bs16101868

**Published:** 2026-10-10

**Authors:** Mónica Bravo-Sanzana, Matías Chávez-Contreras, Rafael Miranda, Xavier Oriol, Jorge Varela, Manuel Mieres-Chacaltana

**Affiliations:** 1 Núcleo Científico-Tecnológico en Ciencias Sociales y Humanidades, Universidad de La Frontera, Temuco 4780000, Chile; 2 Departamento de Ingeniería Industrial y de Sistemas, Universidad de La Frontera, Temuco 4780000, Chile; matias.chavez@ufrontera.cl; 3 Departamento de Psicología, Universidad de Girona, 17004 Girona, Spain; rafael.miranda@udg.edu (R.M.); xavier.oriol@udg.edu (X.O.); 4 Facultad de Psicología, Universidad del Desarrollo, Santiago 7610658, Chile; jovarela@udd.cl; 5 Departamento de Diversidad y Educación Intercultural, Facultad de Educación, Universidad Católica de Temuco, Temuco 4781312, Chile; mieres@uct.cl

**Keywords:** school social climate, educational assistants, ecological perspective, exploratory graph analysis, psychometric evaluation, educational coexistence

## Abstract

Educational assistants remain underrepresented in multidimensional assessments of school social climate. This study developed the Multidimensional School Social Climate Inventory for Educational Assistants (MSSCI-EA) and examined initial evidence regarding its content validity, internal structure, and internal consistency. Instrument development involved a systematic review, item generation, expert judgment, and pilot testing. The main study included 871 educational assistants from 91 Chilean schools. Exploratory Graph Analysis (EGA) was conducted using Spearman correlations, GLASSO network estimation, and Louvain community detection. Internal consistency was estimated using Cronbach’s alpha and McDonald’s omega based on Pearson correlations. Item stability was examined through 1000 bootstrap resamples, and hierarchical EGA assessed whether a unidimensional higher-order structure could support an overall score. The retained solution comprised 90 items across 12 dimensions. Both alpha and omega ranged from 0.773 to 0.957. Average item stability, calculated according to the final dimension assignments, ranged from 0.252 to 1.000. Hierarchical EGA identified five higher-order communities and did not support a unidimensional interpretation of an overall total score. These findings provide initial psychometric evidence for the MSSCI-EA. Further evaluation of the less stable item groupings and independent confirmation of the dimensional structure are needed.

## 1. Introduction

### 1.1. School Social Climate as an Ecological and Multidimensional Construct

School social climate is one of the most relevant constructs for understanding the functioning of educational communities because of its consistent association with multiple indicators of individual and organizational development. Over the past several decades, accumulating evidence has shown that the perceptions developed by members of the educational community regarding interpersonal relationships, safety, school norms, pedagogical practices, and institutional conditions are systematically associated with well-being, mental health, school engagement, academic achievement, job satisfaction, and educational coexistence (Aldridge & McChesney, 2018; Cohen et al., 2009; Korpershoek et al., 2020; Thapa et al., 2013). Consequently, school social climate has evolved from being regarded as a secondary contextual attribute to becoming a central component for understanding the quality of educational processes and promoting the holistic development of those who participate in school life.

In this study, school social climate emphasizes the social experiences examined within the broader school climate literature. Belonging, interpersonal relationships, and safety are components of this multidimensional construct, while organizational and working conditions are included insofar as they shape how educational assistants experience everyday school life (Cohen et al., 2009; Grazia & Molinari, 2021; Rudasill et al., 2018; Wang & Degol, 2016). Within this multidimensional conception, interpersonal relationships, school connectedness and belonging, safety, and institutional conditions represent interrelated components of the broader school social climate rather than isolated domains of school experience. Their configuration reflects the ways in which educational actors experience the school environment through their interactions, roles, and participation in school life.

One of the most influential conceptual frameworks for organizing this complexity was proposed by Wang and Degol (2016), who synthesized the available evidence into four domains (Academic, Community, Safety, and Institutional Environment) encompassing thirteen dimensions that capture the principal characteristics of school social climate described in the international literature. This framework represents one of the most comprehensive conceptual taxonomies developed to date and has served as the foundation for numerous assessment instruments. Nevertheless, the authors acknowledge that the boundaries between some dimensions are not always clearly distinguishable and that their framework represents a conceptual synthesis derived from the available literature rather than a psychometric structure that is necessarily invariant across populations or educational contexts (Wang & Degol, 2016).

This interpretation is consistent with Bronfenbrenner and Morris’s (2006) bioecological model of human development, which proposes that individual development and institutional functioning emerge through reciprocal interactions between individuals and the multiple ecological systems in which they participate. Applied to the educational context, this perspective conceptualizes school social climate as an emergent property arising from the interactions among students, teachers, educational assistants, school leadership teams, families, and the broader institutional environment. This ecological perspective provides the basis for considering the experiences of different members of the educational community when assessing school social climate.

In Latin America, research on school social climate has grown considerably over recent decades. Nevertheless, important challenges remain regarding the conceptual diversity of the construct, the heterogeneity of available assessment instruments, and the limited incorporation of multidimensional and multi-informant approaches. A recent systematic review showed that most instruments developed in the region continue to rely on partial assessments of school social climate, frequently focusing on a single educational actor and providing limited psychometric evidence to support their use across different contexts (Bravo-Sanzana et al., 2023). The findings of this review provided the conceptual and methodological foundation for the development of the present instrument by guiding the selection of dimensions, the construction of the initial item pool, and the adoption of an ecological and multidimensional framework that recognizes the relevance of multiple educational perspectives in school social climate assessment.

### 1.2. Towards a Multi-Informant Assessment of School Social Climate

The increasing conceptual complexity attributed to school social climate has highlighted the need to move beyond assessment approaches based on a single informant. If school social climate is understood as a relational phenomenon emerging from the everyday interactions among multiple actors and contextual conditions, its assessment should incorporate the perspectives of individuals who participate in school life from distinct functional roles and responsibilities (Bronfenbrenner & Morris, 2006; Wang & Degol, 2016). From this perspective, the perceptions of students, teachers, school leadership teams, families, and other members of the educational community should not be regarded as redundant measurements of the same phenomenon but rather as complementary sources of information that provide a more comprehensive understanding of the complexity of school processes.

Consistent with this perspective, Wang and Degol (2016) identified the incorporation of multiple informants as one of the principal challenges for advancing research on school social climate. Differences across stakeholders’ perceptions should not necessarily be interpreted as measurement error or as evidence of poor agreement between assessment instruments. Instead, they reflect the fact that each group experiences and interprets the school environment through its own roles, responsibilities, and patterns of interaction. Integrating these diverse perspectives therefore provides a more comprehensive representation of school functioning and facilitates the identification of strengths and areas for improvement that may remain undetected when assessment is restricted to a single group of informants.

Despite this growing conceptual consensus, available evidence indicates that research continues to rely predominantly on the perceptions of students and teachers, whereas other members of the educational community remain substantially underrepresented in the development and validation of school social climate instruments (Bravo-Sanzana et al., 2023; Grazia & Molinari, 2021; Wang & Degol, 2016). This imbalance limits the understanding of school social climate as an ecological phenomenon because it overlooks relational experiences constructed from different institutional roles and constrains the ability to examine how diverse groups perceive and experience the same educational context.

To address these limitations, the Multidimensional School Social Climate Inventory (MSSCI) project was established to develop actor-specific assessment instruments within a common conceptual framework grounded in ecological and multi-informant principles. This approach acknowledges that educational actors occupy different functional positions within the school organization and may therefore contribute complementary information for understanding the relational dynamics of educational communities. Within this framework, the progressive development of MSSCI versions for students and other members of the educational community represents an important step toward a more comprehensive assessment of school social climate, consistent with the multidimensional nature of the construct and with the priorities identified in the international literature (Bravo-Sanzana et al., 2023, 2025).

### 1.3. Educational Assistants: An Underrepresented Perspective in School Social Climate Research

Educational assistants remain underrepresented in multidimensional assessments of school social climate in Latin America (Bravo-Sanzana et al., 2023). School climate instruments have primarily examined student and teacher perspectives; the MSSCI-A is one example of an actor-specific instrument for adolescents (Bravo-Sanzana et al., 2025). The gap addressed here concerns the limited representation of educational assistants in multidimensional assessment, rather than an absence of school climate measures for other groups. To date, most studies have focused primarily on the perceptions of students and teachers, whereas the participation of other members of the educational community has remained limited, despite their essential contributions to the daily functioning of schools and to the development of safe, inclusive, and supportive learning environments (Bravo-Sanzana et al., 2023; Grazia & Molinari, 2021; Wang & Degol, 2016).

Within the international literature, research has concentrated largely on professionals identified as teaching assistants, paraprofessionals, and school support staff, whose roles have been examined predominantly in the context of inclusive education. Masdeu Navarro (2015) reviewed the roles and responsibilities of learning support staff, defined in that report as personnel whose main function is to assist teachers. A systematic review conducted by Sharma and Salend (2016) demonstrated that these professionals perform responsibilities extending well beyond administrative or logistical support, actively contributing to classroom management, instructional support, behavior regulation, socio-emotional assistance, and the facilitation of participation among students with diverse educational needs. However, the authors also emphasized that role ambiguity, limited specialized training, and insufficient coordination with teachers may hinder the effective performance of these responsibilities, thereby affecting both the quality of inclusive education and overall school functioning.

In a qualitative study involving semi-structured interviews in four Welsh secondary schools, Littlecott et al. (2018) examined how support staff and teachers contributed to relationships with students and to the provision of well-being support. Their findings describe opportunities for support staff to build trust and provide individualized assistance alongside teaching staff. As a result, students may perceive them as an additional source of support for addressing personal and socio-emotional concerns. These findings suggest that educational assistants may contribute complementary perspectives on school social climate that enrich the understanding of relational processes within educational communities.

Recent research has begun to document occupational challenges experienced by school paraprofessionals. In a U.S. study involving 1993 paraprofessionals, Reddy et al. (2024) examined their experiences of student-perpetrated violence and the relationships of those experiences with student–staff interactions and student behavioral concerns. This evidence draws attention to aspects of safety and everyday school relationships that merit consideration when examining educational assistants’ perceptions of school social climate.

In Chile, educational assistants constitute a legally recognized professional group under Law No. 21,109, which regulates their professional development and defines responsibilities related to pedagogical support, institutional management, educational coexistence, inclusion, student support, and the administrative and operational functioning of schools. Although this professional category is not fully equivalent to those described in the international literature, it shares the defining characteristic of playing a continuous role in shaping the everyday relationships that configure school life. This perspective is further supported by growing evidence highlighting the importance of understanding the well-being, resilience, prosocial behavior, and relational processes of adults working within educational communities as essential components of organizational functioning and healthy school environments (Salvo-Garrido et al., 2024; Mieres-Chacaltana et al., 2025a, 2025b). Nevertheless, despite their relevance within the Chilean educational system, educational assistants remain underrepresented in multidimensional school social climate assessment. The systematic review underpinning the present study identified important limitations in the inclusion of multiple educational actors in existing measures, providing the basis for developing an actor-specific instrument for educational assistants within a common multidimensional framework (Bravo-Sanzana et al., 2023).

### 1.4. Conceptual and Methodological Challenges in Assessing School Social Climate

Developing actor-specific measures of school social climate also raises an important methodological question: whether its conceptual dimensions are empirically organized in the same way across educational groups. If school social climate is understood as a phenomenon emerging from multiple relational experiences, its empirical structure should not necessarily be expected to be identical across the different members of the educational community. Variations in professional roles, institutional responsibilities, and everyday interactions may give rise to distinct configurations of the construct without altering its underlying conceptual foundation. Rather, these differences reflect alternative ways in which a shared educational reality is experienced and interpreted (Bronfenbrenner & Morris, 2006; Grazia & Molinari, 2021; Wang & Degol, 2016).

This complexity has also been acknowledged within the school social climate literature. Although the framework proposed by Wang and Degol (2016) currently represents one of the most comprehensive conceptual models for organizing the construct, the authors themselves recognize that several dimensions exhibit considerable conceptual overlap and that empirical evidence regarding the dimensional organization of school social climate remains heterogeneous across educational settings and populations. Consequently, evaluating the internal structure of instruments designed for specific educational actors requires analytical procedures capable of examining how these dimensions are empirically organized rather than imposing an a priori factorial structure.

Within this context, Exploratory Graph Analysis (EGA) has emerged as a methodological approach grounded in network psychometrics for investigating the dimensionality of complex psychological constructs. Unlike traditional latent variable approaches, EGA represents items as a network of regularized partial correlations and identifies communities of highly interconnected indicators, thereby allowing researchers to examine the dimensional structure of a construct directly from the observed pattern of relationships among items (H. F. Golino & Epskamp, 2017; H. Golino et al., 2020). From this perspective, dimensions are not specified in advance by the analytical model but instead emerge empirically from the relational structure of the data.

The application of EGA is particularly appropriate for examining how the conceptual organization of school social climate is empirically configured within a specific educational actor group, because it allows researchers to determine whether the dimensions proposed in the literature are maintained or reorganized when examined from a particular functional position within the school community. Accordingly, this approach provides a methodological strategy that is closely aligned with the relational nature of school social climate and with the objective of developing assessment instruments capable of accurately representing the perspectives of diverse members of the educational community. Because the primary objective of the present study was to identify how school social climate is empirically organized from the perspective of educational assistants, rather than to test a previously established factorial structure, Exploratory Graph Analysis (EGA) was selected as the principal analytical strategy. This approach is therefore consistent with the ecological framework of the present instrument and with the broader multi-informant framework of the MSSCI project.

### 1.5. The Present Study

Building upon the conceptual, empirical, and methodological foundations outlined above, the present study aimed to develop the Multidimensional School Social Climate Inventory for Educational Assistants (MSSCI-EA) and provide initial psychometric evidence in a sample of educational assistants working in Chilean schools. Specifically, the study addressed four components of instrument development and evaluation: (1) development of the item pool based on the multidimensional framework proposed by Wang and Degol (2016) and the systematic review underpinning the MSSCI project; (2) assessment of content validity evidence through expert judgment and pilot testing; (3) examination of the instrument’s internal structure using Exploratory Graph Analysis (EGA); and (4) evaluation of internal consistency using Cronbach’s alpha and McDonald’s omega coefficients.

The MSSCI-EA was developed within a broader ecological and multi-informant framework while focusing specifically on educational assistants as the informant group examined in the present study. Correlations among the empirically identified dimensions were also examined to characterize their pattern of associations.

## 2. Materials and Methods

### 2.1. Study Design

The present study was an instrumental psychometric investigation aimed at the development and initial psychometric evaluation of the Multidimensional School Social Climate Inventory for Educational Assistants (MSSCI-EA). The study was conducted in accordance with established guidelines for the development and evaluation of measurement instruments in the social and behavioral sciences, integrating multiple sources of validity evidence, including conceptual item development, content validity evaluation through expert judgment, and analyses of the instrument’s internal structure and internal consistency (American Educational Research Association et al., 2014; Muñiz & Fonseca-Pedrero, 2019).

The MSSCI-EA was developed within the broader ecological and multi-informant framework of the MSSCI project. However, the present study examined a single informant group—educational assistants—and the psychometric evidence reported here pertains specifically to their responses. The instrument was designed to assess educational assistants’ perceptions of school social climate while maintaining a common conceptual framework with MSSCI versions developed for other members of the educational community. The present study did not examine empirical equivalence or psychometric comparability between the MSSCI-EA and other actor-specific MSSCI versions. At the same time, the instrument was specifically adapted to reflect the distinctive functional roles and experiences of educational assistants within school life.

### 2.2. Instrument Development

The development of the Multidimensional School Social Climate Inventory for Educational Assistants (MSSCI-EA) was informed by a systematic review of school social climate instruments used in Latin America, conducted in accordance with the PRISMA guidelines. This review identified the principal conceptual approaches, dimensions, and indicators reported in the literature, while also highlighting important limitations regarding the multidimensional coverage of the construct, the inclusion of multiple informants, and the available psychometric evidence (Bravo-Sanzana et al., 2023).

The conceptual framework proposed by Wang and Degol (2016) served as the theoretical foundation for instrument development. Their model comprises four domains—Academic, Community, Safety, and Institutional Environment—encompassing thirteen dimensions: Leadership, Teaching and Learning, Professional Development, Quality of Relationships, Connectedness, Respect for Diversity, Community Partnerships, Social and Emotional Safety, Physical Safety, Order and Discipline, Environmental Adequacy, Structural Organization, and Availability of Resources. Drawing upon the instruments and indicators identified in the systematic review by Bravo-Sanzana et al. (2023), the initial items were formulated and organized according to the dimensions proposed by Wang and Degol (2016) through a committee-based procedure involving two independent specialists in psychological measurement and assessment and one member of the research team.

Although the actor-specific instruments developed within the MSSCI project share a common conceptual framework, their item pools were adapted to the characteristics and informational scope of each educational actor. In particular, the Professional Development dimension was considered relevant for adults working within schools, including teachers, educational assistants, and school leadership teams. Likewise, only those indicators that could be meaningfully and validly evaluated from the functional perspective of educational assistants were retained and operationalized.

The preliminary version of the MSSCI-EA comprised 116 items, presented in full in Supplementary Table S1. Following the content validity evaluation, the removal or revision of redundant indicators and the separation of several double-barreled items resulted in a revised pool of 115 items, which was subsequently administered in both the pilot study and the main psychometric study. Supplementary Table S2 documents the correspondence between the previous and revised item numbers and wording, item status, and specific changes, including revisions, splits, additions, and removals, in Spanish and English.

Items assessing violence and discrimination were reverse-coded so that higher scores represented more favorable perceptions of school social climate, consistent with the interpretation of the remaining items.

### 2.3. Content Validity Assessment

The preliminary version of the instrument was evaluated by a panel of five experts, all of whom held doctoral degrees in psychology. Three conducted research on school coexistence and school climate, one specialized in measurement and data analysis, and one had expertise in school climate management. Each expert assessed every item in terms of clarity, coherence, and relevance, and also evaluated the adequacy of the proposed set of indicators for representing each theoretical dimension, following the recommendations of Escobar-Pérez and Cuervo-Martínez (2008) and Ventura-León (2022).

Items were rated using a four-point ordinal scale ranging from 0 (criterion not met) to 3 (criterion fully met). In addition, the experts provided qualitative feedback to identify problems related to wording, ambiguity, redundancy, and the inclusion of multiple conceptual contents within a single item. Supplementary Table S1 provides the initial item wording in Spanish and English, together with the mean expert ratings and Aiken’s V coefficients for each item and evaluation criterion.

### 2.4. Pilot Study

The resulting version of the instrument was administered to a pilot sample of 52 educational assistants. Participants were asked to report any usability problems encountered with the online administration platform. Simple descriptive analyses of item responses were also conducted to verify that no item had zero variance.

No platform usability problems were reported, and no items exhibited zero response variance. Consequently, the 115-item version was retained without further modifications and subsequently administered in the main study.

### 2.5. Instrument

The Multidimensional School Social Climate Inventory for Educational Assistants (MSSCI-EA) was developed to assess educational assistants’ perceptions of school social climate within the ecological and multidimensional framework of the broader MSSCI project. The version administered in the present study consisted of 115 items, initially organized according to the conceptual framework proposed by Wang and Degol (2016), which comprises four domains and thirteen dimensions of school social climate.

Items were specifically written to represent situations, interpersonal relationships, and institutional conditions that educational assistants could reasonably observe and experience while performing their professional responsibilities within the school community. All items were rated using a five-point Likert-type scale, with higher scores indicating more favorable perceptions of school social climate.

The final dimensional structure of the instrument was subsequently examined using Exploratory Graph Analysis (EGA), which enabled the empirical identification of the underlying organization of the indicators from the perspective of educational assistants.

### 2.6. Participants

The main study sample comprised 871 educational assistants working in Chilean schools. The participants worked in 91 schools, identified by unique school identification codes (RBD). Participants represented a broad range of support roles within educational communities, reflecting the functional diversity established for this professional group within the Chilean educational system.

Participation was voluntary and contingent upon the provision of informed consent. The sample used for the psychometric analyses was independent of the 52 educational assistants who had participated in the pilot study.

Because the primary objective of the study was to examine the internal structure of the instrument from the specific perspective of educational assistants, the entire sample was retained for the Exploratory Graph Analysis (EGA). The sample was not divided into exploratory and confirmatory subsamples, as preserving the full dataset was considered methodologically preferable for obtaining a more stable and precise estimation of the network structure underlying the associations among items.

Participant and school characteristics are summarized in Table 1.

### 2.7. Procedure

Data collection was conducted in Chilean schools that voluntarily agreed to participate in the study. Initial contact with participating institutions was established through a nationwide recruitment strategy targeting school leadership teams and professionals responsible for educational coexistence. During this stage, information was provided regarding the objectives of the study, the characteristics of the instruments, the administration procedures, and the ethical safeguards governing data collection and management (Scientific Ethics Committee Approval N° 046/22).

Schools that agreed to participate formalized their involvement through institutional authorization and designated a local coordinator to liaise with the research team throughout the data collection process. Before administering the instruments, coordinators received standardized technical guidance regarding participant eligibility criteria, administration procedures, response instructions, the voluntary nature of participation, and the measures implemented to ensure confidentiality.

Educational assistants were invited to complete the questionnaire voluntarily and anonymously. Before accessing the online survey, participants received detailed information regarding the purpose of the study, the exclusive scientific use of the collected data, the absence of any consequences associated with the decision to participate or decline participation, and their right to withdraw from the study at any stage without penalty. Participation proceeded only after informed consent had been provided.

The questionnaire was administered through QuestionPro (https://www.questionpro.com/es/), with procedures designed to ensure individual completion. Responses were stored in a protected database and analyzed exclusively in aggregated form. The study was conducted in accordance with internationally recognized ethical principles for research involving human participants and received approval from the corresponding Scientific Ethics Committee.

### 2.8. Data Analysis

For the content validity evaluation, item adequacy was determined using two complementary criteria: a mean expert rating greater than 2.0 and an Aiken’s V coefficient exceeding 0.70. These criteria were used to guide decisions regarding item retention, revision, or elimination before the pilot study.

The internal structure of the MSSCI-EA was examined using Exploratory Graph Analysis (EGA), a network psychometric approach that identifies empirical dimensions based on the patterns of associations among items (H. F. Golino & Epskamp, 2017; H. Golino et al., 2020). Given the ordinal nature of the response scale, the EGA was conducted using a Spearman correlation matrix.

The association network was estimated from the Spearman correlation matrix using the Graphical Least Absolute Shrinkage and Selection Operator (GLASSO; Friedman et al., 2008), which regularizes the partial correlations among items to obtain a sparse and parsimonious network structure. Item communities were subsequently identified using the Louvain community detection algorithm (Blondel et al., 2008), which partitions the network into groups of indicators that are more strongly connected to one another than to the remaining nodes (H. F. Golino & Epskamp, 2017; H. Golino et al., 2020).

Item retention and exclusion in the EGA stage were determined using a loading-based statistical criterion. Items were retained when their highest loading across the identified communities was at least 0.30 and were assigned to the community corresponding to that loading. Items with a maximum loading below 0.30 were excluded. Cross-loadings were defined as loadings greater than 0.30 on two or more dimensions. Items exhibiting cross-loadings would be retained only if their primary loading was at least twice their second-highest loading; otherwise, they would be excluded. Conceptual review informed the interpretation and labeling of the resulting dimensions rather than constituting an additional criterion for item exclusion at this stage. The complete loading matrix, including primary and secondary loadings, is presented in Supplementary Table S3. Internal consistency was subsequently estimated for each dimension using Cronbach’s alpha and McDonald’s omega coefficients. Finally, Spearman correlations among the retained dimensions were computed to examine the degree of association between the different components of school social climate represented by the instrument. The analyses were conducted in R version 4.6.0 (R Core Team, 2026) using RStudio version 2026.8.2.200 (Posit Team, 2026). EGA, including network estimation and Louvain community detection, was performed using EGAnet version 2.4.1 (H. Golino & Christensen, 2026), and internal consistency estimates were obtained using psych version 2.6.5 (Revelle, 2026).

Item stability was examined using bootstrap EGA (bootEGA) with 1000 iterations and the resampling method applied to the 90 retained items. Each bootstrap iteration used Spearman correlations, GLASSO network estimation, and Louvain community detection, consistent with the EGA procedure. A fixed random seed of 123 was used for the bootstrap analysis to ensure computational reproducibility. Item stability was defined as the proportion of bootstrap samples in which an item was assigned to its corresponding empirical dimension. For descriptive reporting, these item-level proportions were averaged according to the final item allocation to each of the 12 dimensions. Resampling provides evidence of stability within the available sample and does not constitute independent confirmation of the dimensional structure.

A hierarchical EGA was also conducted to examine whether a unidimensional higher-order structure could support the interpretation of an overall score. The higher-order network was estimated using Spearman correlations and GLASSO with EBIC model selection (gamma = 0.50). Community detection used the Louvain algorithm with the most common solution across 1000 consensus iterations, and unidimensionality was assessed using the Louvain method.

## 3. Results

### 3.1. Content Validity Evidence

The content validity evaluation showed satisfactory evidence of clarity (M = 2.88; V = 0.98), coherence (M = 2.68; V = 0.93), and relevance (M = 2.80; V = 0.97). Based on the quantitative ratings and qualitative feedback, of the 116 original items, 17 items were eliminated, 8 double-barreled items were divided into 18 conceptually distinct statements, and 6 new items were added based on the experts’ recommendations. This process resulted in a 115-item version, which was subsequently evaluated in the pilot study. The complete content review and item modifications are presented in Supplementary Tables S1 and S2.

The pilot study identified no reported usability problems with the online administration platform. Descriptive analyses confirmed that all items had nonzero response variance. Therefore, the 115-item version was retained without further modification and was subsequently administered in the main study.

### 3.2. Internal Structure Evidence

The internal structure of the Multidimensional School Social Climate Inventory for Educational Assistants (MSSCI-EA) was examined using Exploratory Graph Analysis (EGA). The retained solution comprised 90 items assigned to 12 empirical dimensions. This solution represents an exploratory configuration identified in the present sample, rather than an established psychometric structure. Its interpretation should take into account the differences in item stability reported in Section 3.5.

The twelve dimensions identified were Emotional Safety, Order and Discipline, Respectful and Nonviolent School Coexistence, Violence and Discrimination, Participation and Collaboration, Relationships within the School Community, Sense of Belonging, Leadership and Professional Development, Respectful Treatment by Teachers, Physical and Classroom Conditions, Laboratory Resources, and Material and Human Resources.

Table 2 presents the dimensional structure identified through EGA, including the number of retained items and the range of loadings observed for each dimension. Supplementary Table S3 reports the EGA loadings of all 115 analyzed items across the 12 communities, with item wording in Spanish and English. Applying the loading criterion resulted in the retention of 90 items and the exclusion of 25 items, each of which had a maximum loading below 0.30. Among the retained items, none had a secondary loading of 0.30 or higher. The largest secondary loading was 0.120, and the smallest difference between the two highest loadings was 0.221. These descriptive results indicate that none of the retained items had closely competing primary and secondary loadings in the present solution. Supplementary Table S4 identifies the 90 retained items, their item codes, final dimension assignments, wording in Spanish and English, and primary loadings. Supplementary Table S5 lists the 25 excluded items and their wording in Spanish and English; their loadings across all communities are reported in Supplementary Table S3. Retention decisions were based on the unrounded loading values.

### 3.3. Internal Consistency Evidence

The internal consistency of the twelve dimensions identified through Exploratory Graph Analysis (EGA) was estimated using Cronbach’s alpha and McDonald’s omega coefficients based on Pearson correlations. Both Cronbach’s alpha and McDonald’s omega values ranged from 0.773 to 0.957. Across most dimensions, both reliability coefficients yielded highly similar estimates. Complete reliability estimates are presented in Table 3.

### 3.4. Relationships Among the Dimensions

The correlations among the twelve dimensions identified through Exploratory Graph Analysis (EGA) revealed predominantly positive associations of varying magnitude across the different components of school social climate.

Table 4 presents the complete correlation matrix among the identified dimensions, together with their corresponding levels of statistical significance.

### 3.5. Item Stability Across Bootstrap Samples

Item stability was examined using 1000 bootstrap samples generated by resampling. Across the 90 retained items, the proportions of replication in their empirical dimensions ranged from 0.186 to 1.000. Average item stability was calculated for each of the 12 final dimensions by grouping the item-level proportions according to the final allocation reported in Supplementary Table S4. These averages ranged from 0.252 to 1.000. Respectful and Nonviolent School Coexistence had an average item stability of 0.252, and Physical and Classroom Conditions had an average of 0.678. The remaining ten dimensions had averages ranging from 0.982 to 1.000. Table 5 presents the average item stability for each final dimension, and Supplementary Table S6 reports the individual item values.

### 3.6. Higher-Order Structure and Unidimensionality

A hierarchical exploratory graph analysis was conducted to assess whether a unidimensional higher-order structure could support the calculation of an overall MSSCI-EA score. The higher-order network was estimated using Spearman correlations and GLASSO, with EBIC model selection. Community detection used the Louvain algorithm with the most common solution across 1000 consensus iterations. The higher-order network yielded five communities. The Louvain unidimensionality assessment did not identify a unidimensional solution. Thus, this analysis did not provide evidence for a single higher-order dimension that would justify interpreting an overall total score as a unidimensional measure.

## 4. Discussion

### 4.1. Internal Structure of the MSSCI-EA

The empirical configuration identified through Exploratory Graph Analysis (EGA) revealed a partial reorganization of the dimensions originally proposed by Wang and Degol (2016). The resulting dimensions offer an interpretable account of school social climate as reported by educational assistants, bringing together relational, safety-related, and institutional aspects of school life. The ecological perspective provides a useful framework for interpreting this configuration in relation to the everyday interactions and responsibilities of educational actors (Bronfenbrenner & Morris, 2006). These findings contribute to the development of assessment instruments that incorporate educational assistants’ experiences within a broader multidimensional understanding of school social climate.

Despite this reorganization, the empirical solution retained clear conceptual correspondence with the multidimensional framework proposed by Wang and Degol (2016). Emotional Safety, Order and Discipline, and Violence and Discrimination remained closely aligned with the safety-related components of the framework; Relationships within the School Community, Sense of Belonging, Participation and Collaboration, and Respectful Treatment by Teachers reflected its relational and community-oriented components; and Physical and Classroom Conditions, Laboratory Resources, and Material and Human Resources retained the institutional-environmental emphasis of the original model. Respectful and Nonviolent School Coexistence encompassed items addressing the value of school rules, nonviolent conflict resolution, and respect for differences, connecting safety-related and relational aspects of school life. Leadership and Professional Development emerged as a combined dimension, illustrating how conceptually related components of the original framework may be reorganized when school social climate is examined from the perspective of educational assistants. At the same time, the observed reorganization is consistent with the possibility that functional position may contribute to how school social climate is experienced and organized by different members of the educational community. Direct comparison across actor groups will be necessary to examine this possibility empirically. These findings therefore highlight the value of incorporating actor-specific perspectives within broader multi-informant approaches to school social climate research (Bronfenbrenner & Morris, 2006; Wang & Degol, 2016).

### 4.2. Educational Assistants as a Distinct Source of Information on School Social Climate

One of the principal contributions of this study is the recognition of educational assistants as a distinct source of information on school social climate rather than simply as an occupational group to whom instruments originally developed for teachers can be applied. Although school social climate research has progressively incorporated multiple educational actors, empirical evidence continues to focus primarily on students, teachers, and families, while the experiences of educational assistants remain substantially underrepresented in both theoretical models and the development of psychometric instruments (Wang & Degol, 2016).

From an ecological perspective, this distinction is conceptually meaningful. Educational assistants occupy a unique institutional role within the school organization, participating daily in student support, supervision, pedagogical assistance, educational coexistence, psychosocial services, administrative tasks, and institutional management. Consequently, their interactions, responsibilities, and lived experiences provide a distinctive perspective on school social climate that complements—but is not equivalent to—that of teachers, students, or families (Bronfenbrenner & Morris, 2006).

A qualitative study in four Welsh secondary schools offers an illustration of this distinction. Littlecott et al. (2018) suggested that school support staff may establish forms of interaction with students that complement those developed by teachers, particularly by providing opportunities for individualized support outside routine classroom activities. Although the category of school support staff examined by Littlecott et al. (2018) is not fully equivalent to educational assistants as defined in the Chilean educational system, the study nevertheless highlights the value of incorporating the perspectives of professionals who support students through roles distinct from classroom teaching. Similarly, in their systematic review of teaching assistants in inclusive educational settings, Sharma and Salend (2016) concluded that these professionals perform pedagogical, relational, and educational coexistence responsibilities that extend well beyond the traditional definitions of their roles, highlighting the need to understand their experiences through conceptual frameworks specifically tailored to their professional context.

The present study extends this body of research by providing initial psychometric evidence for an instrument developed specifically for educational assistants. The empirical configuration retained several core components described in the school climate literature while organizing them into dimensions with meaningful content for this group. These findings support the relevance of including educational assistants in multidimensional school climate assessment and provide a basis for further investigation of their perceptions within the broader MSSCI project.

From this standpoint, functional position provides a relevant conceptual lens for interpreting how educational assistants experience school social climate. Their roles, responsibilities, and patterns of interaction may shape the aspects of school life that are most salient from their perspective. The actor-specific configuration identified in the present study is consistent with this interpretation; however, determining whether the empirical organization of school social climate differs systematically across professional roles requires direct comparison among educational actor groups.

### 4.3. School Social Climate, Organizational Well-Being, and Relational Resources

The identified dimensions encompass relational and organizational aspects of school life that are relevant to the broader literature on well-being in educational communities. Emotional Safety, Relationships within the School Community, Leadership and Professional Development, and Respectful Treatment by Teachers give these aspects an explicit place within the assessment of educational assistants’ perceptions of school social climate. Although associations with well-being indicators were not examined in this study, these dimensions provide a basis for investigating such relationships in future research.

Studies involving Chilean elementary school teachers have examined associations among resilience, prosocial behavior, and self-efficacy (Mieres-Chacaltana et al., 2025a, 2025b). These studies provide a conceptual basis for considering personal and relational resources when investigating adults’ experiences in educational communities. Future research could examine how these resources relate to educational assistants’ perceptions of school social climate and their well-being.

From this perspective, the configuration identified through EGA brings relational experiences into focus alongside environmental and disciplinary conditions. Collaboration, support, and recognition are relevant areas for understanding the everyday experiences of educational assistants, whose responsibilities involve interaction with students, teachers, school leadership teams, and families. Examining how these aspects of perceived school social climate relate to occupational and psychological well-being represents a valuable direction for extending the present findings.

The Violence and Discrimination dimension and the availability of psychological and social support captured within Material and Human Resources also draw attention to safety and support needs within the school community. Reddy et al. (2024) documented physical assault and verbal aggression or threats directed at school paraprofessionals, and examined how these experiences relate to student–staff relationships and student behavioral concerns. These findings support the relevance of considering educational assistants’ perspectives when assessing school social climate. Whether their responses provide information distinct from measures designed for other educational actors requires direct comparison in future research.

Taken together, the dimensions identified in this study and the literature discussed above provide a rationale for future research linking educational assistants’ perceptions of school social climate with well-being. Such research could assess which relational and organizational dimensions are associated with well-being indicators and clarify the potential contribution of the MSSCI-EA to this field.

### 4.4. Methodological Contribution and Future Directions for the MSSCI-EA

From a methodological perspective, the MSSCI-EA illustrates an actor-specific approach to school social climate assessment within the common conceptual framework of the MSSCI project. The present findings pertain specifically to educational assistants and do not establish empirical equivalence or psychometric comparability with MSSCI versions developed for other educational actors.

Second, the use of Exploratory Graph Analysis (EGA) made it possible to identify the empirical structure of the instrument without imposing a predetermined factorial organization. Unlike traditional confirmatory approaches, network psychometrics explores how indicators are organized through their partial associations, facilitating the identification of empirical item communities that more faithfully represent the underlying structure of the construct within a specific population (H. F. Golino & Epskamp, 2017; H. Golino et al., 2020). In the present study, this approach identified a twelve-dimensional configuration that retained clear conceptual links with the framework proposed by Wang and Degol (2016) and organized educational assistants’ responses into substantively interpretable domains of school social climate.

In the final 12-dimensional solution, Cronbach’s alpha and McDonald’s omega coefficients both ranged from 0.773 to 0.957. Reporting omega alongside alpha provides a complementary estimate of internal consistency and avoids relying on alpha alone (McNeish, 2018). These estimates describe internal consistency within the retained dimensions; the bootstrap item stability results address a different aspect of the empirical organization and varied across the final item groupings.

This distinction is particularly relevant to Respectful and Nonviolent School Coexistence and Physical and Classroom Conditions. Both dimensions have theoretically interpretable content and showed adequate internal consistency in the present sample (α = 0.831 and ω = 0.834, and α = 0.815 and ω = 0.819, respectively). However, their average item stability proportions (0.252 and 0.678, respectively) indicate that the allocation of their constituent items was less consistent across bootstrap samples. Thus, adequate internal consistency should not be taken as evidence that these item groupings will necessarily emerge as distinct dimensions in independent samples. The bootstrap findings make alternative configurations plausible, including the reassignment of some items to other dimensions.

The provisional retention of Respectful and Nonviolent School Coexistence and Physical and Classroom Conditions preserves conceptually relevant content within the exploratory solution; it does not establish that their items form replicable, distinct dimensions. Their content addresses school rules, nonviolent conflict resolution, respect for differences, and physical conditions, all of which are relevant to established conceptual accounts of school climate (Thapa et al., 2013; Wang & Degol, 2016). However, conceptual relevance and adequate internal consistency do not resolve the uncertainty concerning their dimensional boundaries. The reported average item stability values summarize item-level replication proportions and should not be interpreted as probabilities of reproducing either complete dimensions or the full 12-dimensional configuration. Accordingly, these two item groupings remain provisional and require particular scrutiny in independent samples, including evaluation of alternative item allocations and refinement of indicators where necessary.

These findings expand the availability of actor-specific instruments for assessing school social climate from the perspectives of different members of the educational community. The MSSCI-EA contributes to the advancement of multi-informant approaches by systematically incorporating the experiences of educational assistants into school social climate research. Future studies may examine the convergence and complementarity of different educational actors’ perceptions using instruments developed within comparable conceptual frameworks.

The MSSCI-EA may provide a useful basis for further research on school social climate from the perspective of educational assistants. As additional evidence accumulates regarding its internal structure, reliability, and relations with external variables, future research may also examine its potential contribution to broader school climate assessment processes.

### 4.5. Limitations and Future Research

First, the internal structure of the MSSCI-EA was identified using Exploratory Graph Analysis (EGA), and therefore the proposed dimensional solution should be considered exploratory. Future studies should examine the replicability of this structure in independent samples and evaluate it using confirmatory techniques to determine its stability and generalizability across different educational contexts. Such evidence would provide stronger support for the proposed dimensional structure and further strengthen the psychometric evidence for the MSSCI-EA.

Second, average item stability was lower for Respectful and Nonviolent School Coexistence (0.252) and Physical and Classroom Conditions (0.678) than for the remaining dimensions. These findings warrant further examination of the items assigned to these dimensions and caution in interpreting their composition as stable. The reported averages summarize item-level replication proportions grouped according to the final assignments; they do not establish exact replication of complete dimensions or of the full 12-dimensional structure. Independent confirmation remains necessary.

Third, participants were nested within 91 schools, with an average of 9.6 participants per school (range: 1–35). Shared institutional experiences may have introduced dependence among responses. Because the analyses did not separate within-school from between-school variation, the estimated associations and resulting item communities may reflect both sources of variation. The EGA workflow employed in this study did not directly incorporate school-level clustering into dimensionality estimation or bootstrap item stability assessment. The potential influence of this dependence therefore remains unresolved, and the reported stability estimates should not be interpreted as evidence of robustness to school-level clustering. Subsequent confirmatory studies should explicitly account for school membership using methods appropriate for ordinal indicators and the intended level of inference (Hox, 2010; Maas & Hox, 2005). Where supported by the sampling design and model complexity, multilevel measurement models would help distinguish individual perceptions from shared school-level variation and examine whether the dimensional structure is supported at each level. Future recruitment should therefore consider both the number of schools and the number and distribution of participants within schools.

Finally, the MSSCI-EA was developed within the Chilean educational system. Although the dimensions assessed are grounded in a widely adopted international conceptual framework, cross-cultural adaptation and the evaluation of the instrument’s psychometric properties in other Latin American countries will be necessary to determine its applicability across different educational systems and to facilitate comparative research on school social climate.

Future research should also examine the extent to which the empirical structure of school social climate varies according to the professional role of different educational actors and the institutional contexts in which they operate. Such evidence would move the field beyond the separate psychometric evaluation of actor-specific instruments toward the comparative study of the multiple perspectives that shape school social climate, thereby contributing to the further development of ecological and multi-informant models for its understanding and assessment.

Accordingly, the findings reported here should be interpreted as initial psychometric evidence supporting the development of the MSSCI-EA. Further studies employing independent samples, confirmatory approaches, and cross-cultural evaluations will contribute to consolidating the cumulative evidence supporting the instrument.

## 5. Conclusions

The present study developed the Multidimensional School Social Climate Inventory for Educational Assistants (MSSCI-EA) and provided initial evidence regarding its content validity, internal structure, and internal consistency in a sample of educational assistants working in Chilean schools. The retained EGA solution comprised 90 items assigned to 12 empirical dimensions. The findings provide a basis for further instrument refinement and independent evaluation of this provisional 12-dimensional configuration.

By incorporating the perspective of educational assistants, an educational actor that has been underrepresented in school social climate research, the MSSCI-EA extends the range of actor-specific perspectives considered within the broader MSSCI project. Its contribution at this stage lies primarily in providing a basis for further research on how educational assistants perceive the relational, organizational, and institutional conditions of school life.

More broadly, the present study contributes to an ecological and multidimensional approach to school social climate by examining the perspective of educational assistants within the broader MSSCI project. These findings highlight the relevance of developing actor-specific instruments within a common conceptual framework to capture the perspectives of different members of school communities. Empirical comparability across MSSCI versions, however, requires direct examination in future studies.

## Figures and Tables

**Table 1:** Participant and School Characteristics (N = 871).

Characteristic	Value
**School administration**	
Municipal corporation	13.1
Municipal education department (DAEM)	40.9
Private subsidized	28.8
Private fee-paying	0.1
Local Public Education Service (SLEP)	17.1
**School location**	
Urban	81.0
Rural	19.0
**School type**	
Humanistic-scientific (HC)	73.3
Technical-professional (TP)	7.6
Both HC and TP	19.1
**School socioeconomic group**	
Low	33.2
Lower-middle	33.3
Middle	29.0
Upper-middle	4.4
High	0.1
**Age**	
Valid observations	674
Mean (SD)	41.60 (11.02)

**Table 2:** Empirical Dimensional Structure of the MSSCI-EA Identified Through Exploratory Graph Analysis.

Dimension	Number of Items	Minimum Loading	Maximum Loading
Emotional Safety	6	0.372	0.585
Order and Discipline	4	0.403	0.583
Respectful and Nonviolent School Coexistence	7	0.332	0.441
Violence and Discrimination	11	0.361	0.477
Participation and Collaboration	6	0.363	0.459
Relationships within the School Community	9	0.385	0.493
Sense of Belonging	9	0.324	0.540
Leadership and Professional Development	13	0.328	0.553
Respectful Treatment by Teachers	6	0.427	0.596
Physical and Classroom Conditions	6	0.344	0.554
Laboratory Resources	3	0.598	0.639
Material and Human Resources	10	0.342	0.522

**Note.** Only retained items with primary loadings ≥ 0.30 are summarized. EGA = Exploratory Graph Analysis.

**Table 3:** Internal Consistency Estimates for the MSSCI-EA Dimensions.

Dimension	Cronbach’s α	McDonald’s ω
Emotional Safety	0.896	0.899
Order and Discipline	0.824	0.827
Respectful and Nonviolent School Coexistence	0.831	0.834
Violence and Discrimination	0.787	0.820
Participation and Collaboration	0.773	0.773
Relationships within the School Community	0.907	0.908
Sense of Belonging	0.902	0.905
Leadership and Professional Development	0.935	0.936
Respectful Treatment by Teachers	0.918	0.918
Physical and Classroom Conditions	0.815	0.819
Laboratory Resources	0.957	0.957
Material and Human Resources	0.847	0.853

**Table 4:** Inter-Dimension Correlations for the MSSCI-EA.

Dimensions	D01	D02	D03	D04	D05	D06	D07	D08	D09	D10	D11	D12
D01 Emotional Safety	—											
D02 Order and Discipline	0.461 ***	—										
D03 Respectful and Nonviolent School Coexistence	0.563 ***	0.527 ***	—									
D04 Violence and Discrimination	0.292 ***	0.450 ***	0.345 ***	—								
D05 Participation and Collaboration	0.394 ***	0.448 ***	0.469 ***	0.398 ***	—							
D06 Relationships within the School Community	0.538 ***	0.527 ***	0.551 ***	0.477 ***	0.499 ***	—						
D07 Sense of Belonging	0.573 ***	0.500 ***	0.604 ***	0.389 ***	0.464 ***	0.613 ***	—					
D08 Leadership and Professional Development	0.593 ***	0.489 ***	0.664 ***	0.371 ***	0.511 ***	0.568 ***	0.669 ***	—				
D09 Respectful Treatment by Teachers	0.391 ***	0.401 ***	0.494 ***	0.351 ***	0.384 ***	0.490 ***	0.454 ***	0.470 ***	—			
D10 Physical and Classroom Conditions	0.367 ***	0.398 ***	0.428 ***	0.435 ***	0.352 ***	0.469 ***	0.448 ***	0.458 ***	0.386 ***	—		
D11 Laboratory Resources	0.075 *	0.091 **	0.117 ***	0.009	0.127 ***	0.143 ***	0.122 ***	0.146 ***	0.025	0.186 ***	—	
D12 Material and Human Resources	0.289 ***	0.279 ***	0.323 ***	0.203 ***	0.294 ***	0.316 ***	0.335 ***	0.412 ***	0.261 ***	0.491 ***	0.412 ***	—

**Note.** Values below the diagonal are Spearman correlations. * *p* < 0.05. ** *p* < 0.01. *** *p* < 0.001.

**Table 5:** Average Item Stability for the Final MSSCI-EA Dimensions.

Dimension	Number of Items	Average Item Stability
Emotional Safety	6	0.999
Order and Discipline	4	0.982
Respectful and Nonviolent School Coexistence	7	0.252
Violence and Discrimination	11	1.000
Participation and Collaboration	6	0.998
Relationships within the School Community	9	1.000
Sense of Belonging	9	1.000
Leadership and Professional Development	13	0.999
Respectful Treatment by Teachers	6	1.000
Physical and Classroom Conditions	6	0.678
Laboratory Resources	3	0.988
Material and Human Resources	10	0.994

**Note.** Values are arithmetic means of the item stability proportions from 1000 bootstrap samples (resampling), grouped according to the final item allocation in Supplementary Table S4. Item-level values are reported in Supplementary Table S6.

## Data Availability

The raw data supporting the conclusions of this article will be made available by the authors on request.

## Supplementary Materials

The following supporting information can be downloaded at: https://www.mdpi.com/article/10.3390/bs16101868/s1, Table S1. Initial item wording and item-level content validity evidence (mean expert ratings and Aiken’s V coefficients); Table S2. Item revision record, including previous and revised numbering and wording, item status, and specific changes in Spanish and English; Table S3. EGA loadings of all 115 analyzed items across 12 communities; Table S4. Final allocation of the 90 retained items to 12 dimensions; Table S5. The 25 excluded items; Table S6. Bootstrap item stability proportions for the 90 retained items based on 1000 resamples.
